# Fetal haemodynamic assessment in a case of late-onset intrauterine growth restriction by phase contrast MRI and T2 mapping

**DOI:** 10.1186/1532-429X-17-S1-P27

**Published:** 2015-02-03

**Authors:** Meng Yuan Zhu, Sujana Madathil, Steven Miller, Rory Windrim, Christopher Macgowan, John Kingdom, Mike Seed

**Affiliations:** 1 Cardiology, The Hospital for Sick Children, Toronto, ON, Canada; 2 Physiology and Experimental Medicine, The Hospital for Sick Children, Toronto, ON, Canada; 3 Neurology, The Hospital for Sick Children, Toronto, ON, Canada; 4 Department of Medical Biophysics and Medical Imaging, University of Toronto, Toronto, ON, Canada; 5 Paediatrics, University of Toronto, Toronto, ON, Canada; 6 Department of Obstetrics & Gynaecology, Mount Sinai Hospital, Toronto, ON, Canada; 7 Department of Paediatrics and Diagnostic Imaging, University of Toronto, Toronto, ON, Canada; 8 Maternal-Fetal Medicine, Mount Sinai Hospital, Toronto, ON, Canada

## Background

Late-onset intrauterine growth restriction (IUGR) results from the failure of placenta to supply enough nutrients and oxygen to the rapidly growing late gestation fetus [1]. Inaccuracies in ultrasound based late gestational fetal weight estimation and the absence of typical Doppler changes make late-onset IUGR difficult to detect [2]. We were interested in whether new MRI technology incorporating fetal vessel blood flow and oximetry measurement could improve the sensitivity of conventional fetal monitoring.

## Methods

A normal pregnancy was studied at late gestation as part of an IRB approved study. The pulsatility index (PI) in the umbilical artery (UA) and middle cerebral artery (MCA) were measured using Doppler ultrasound. At 34, 36 and 39 week's gestational age (GA), MRI scans were performed using a 1.5T Siemens Avanto. We measured fetal weight (3D SSFP), the flow and oxygen saturations in the major fetal vessels using phase contrast MRI and T2 mapping according to our previously published technique [3]. Fetal oxygen delivery (DO_2_) and consumption (VO_2_) were calculated using a haemoglobin concentration taken from population averages [4]. We also recorded clinical fetal monitoring and placental histopathology results.

## Results

**Figure 1:**
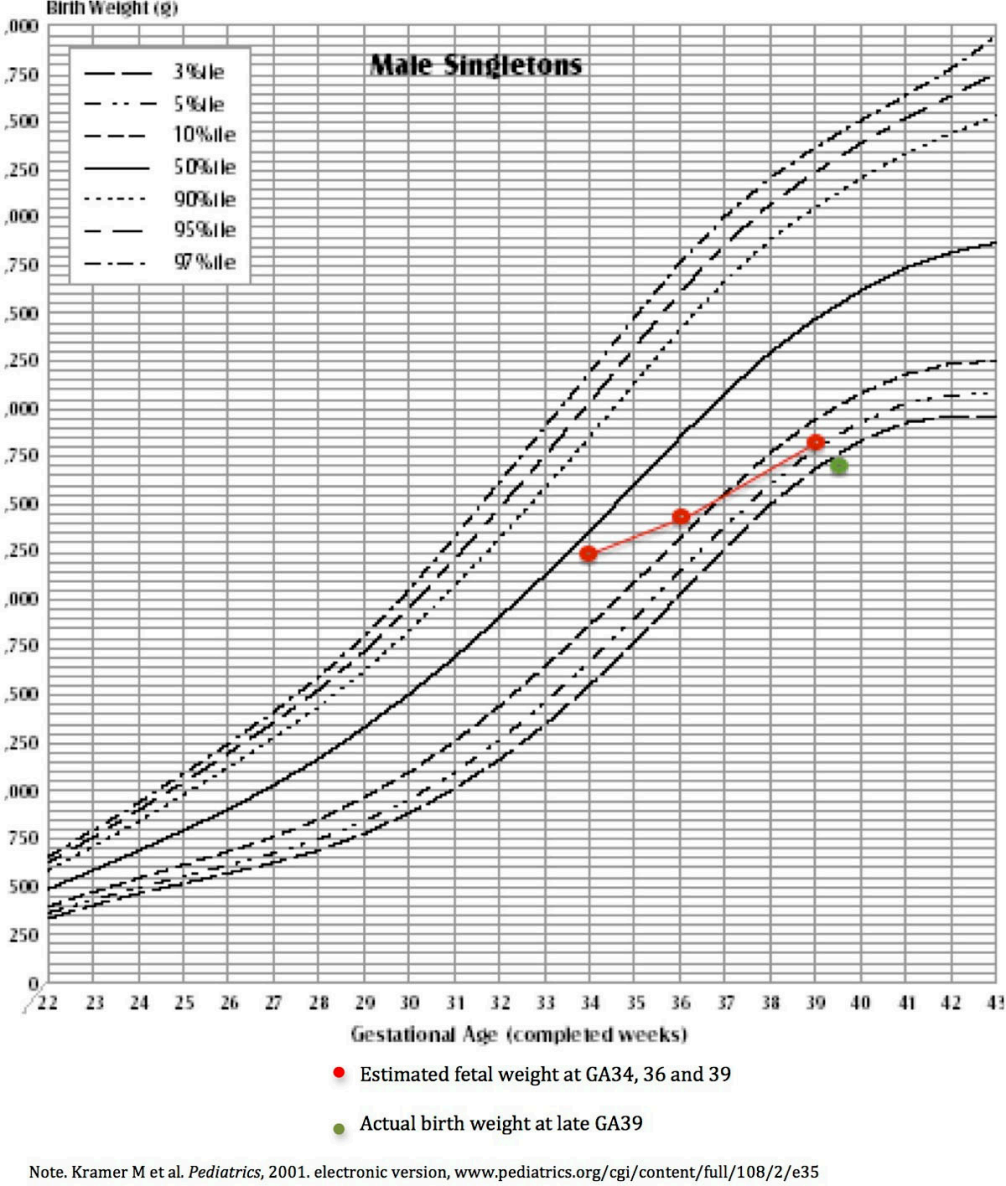
Percentile of estimated fetal weight (GA34: 2.25kg, 38^th^ percentile; GA36: 2.43kg, 17^th^ percentile; GA39: 2.84 8^th^ percentile) and actual birth weight (2.74kg, 5^th^ percentile) when compared with normal fetal population at the same gestation age.

**Figure 2:**
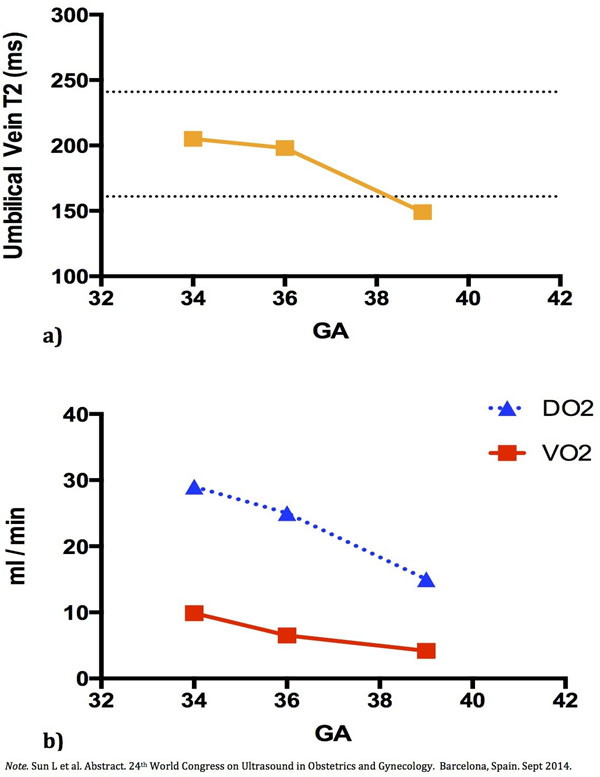
a) Plots of T2 measurements in the umbilical vein at GA34, 36 and 39 comparing with the normal range (Mean T2 ± 2SD). b) Calculated O_2_ consumption (VO_2_) and O2 delivery (DO_2_) at GA34, 36 and 39 based on umbilical vein flows, T2 values of umbilical vein and descending aorta and GA appropriate estimations of hemoglobin concentration.

The UA PI (GA31: 0.9, GA34: 1.0, GA37: 1.1) and MCA PI (GA36: 1.95) were in normal range and there was no clinical suspicion of IUGR at any stage of the pregnancy. Estimated fetal weight increased from 2.25kg to 2.84kg from GA34 to 39; however, the weight percentile dropped from 38^th^ to 8^th^ (Fig. 1). The baby was born in good condition at GA39 weighting 2.74kg (5^th^ percentile). Blood flows in the major fetal vessels were within normal ranges according to our previously published data [5]. Umbilical vein flow decreased as GA increased but within normal range. The T2 values (Fig. 2a), calculated VO2 and DO2 all decreased from GA34 to 39 (Fig. 2b). Placental pathology revealed low normal placental weight (396g: 10^th^ to 25^th^ percentile for GA39) with mild over coiling of the umbilical cord (coiling index: 0.3-0.4) and mild dysmaturity of chorionic villi.

## Conclusions

The fetal weight centile decline, placental histology and small for gestational age birth-weight in this case are in keeping with late-onset IUGR. We propose that the drop in fetal oxygen delivery is evidence of placental insufficiency while the dropped oxygen consumption is in keeping with fetal metabolic adaptation to reduced oxygen delivery, which resulted in slowing of fetal growth. With this novel approach to fetal hemodynamics, MRI could provide clinically significant additional information to conventional fetal monitoring.

## Funding

Canadian Institute of Health Research/Sickkids Foundation New Investigator Award.
